# Myogenic differentiation of primary myoblasts and mesenchymal stromal cells under serum-free conditions on PCL-collagen I-nanoscaffolds

**DOI:** 10.1186/s12896-018-0482-6

**Published:** 2018-11-26

**Authors:** Aijia Cai, Moritz Hardt, Paul Schneider, Rafael Schmid, Claudia Lange, Dirk Dippold, Dirk W. Schubert, Anja M. Boos, Annika Weigand, Andreas Arkudas, Raymund E. Horch, Justus P. Beier

**Affiliations:** 1 Department of Plastic and Hand Surgery and Laboratory for Tissue Engineering and Regenerative Medicine, University Hospital of Erlangen, Friedrich-Alexander University of Erlangen-Nürnberg (FAU), Krankenhausstraße 12, 91054 Erlangen, Germany; 2 Interdisciplinary Clinic for Stem Cell Transplantation, University Cancer Center Hamburg (UCCH), 20246 Hamburg, Germany; 3 Institute of Polymer Materials, Department of Materials Science and Engineering, University of Erlangen-Nürnberg (FAU), Martensstraße 7, 91058 Erlangen, Germany; 4 Department of Plastic Surgery, Hand Surgery, Burn Center University Hospital RWTH Aachen, Aachen, Germany

**Keywords:** Myogenic differentiation, Nanoscaffolds, Electrospun PCL-collagen I-nanofibers, Mesenchymal stromal cells, ADSC, BMSC, Myoblasts, Serum-free media

## Abstract

**Background:**

The creation of functional skeletal muscle via tissue engineering holds great promise without sacrificing healthy donor tissue. Different cell types have been investigated regarding their myogenic differentiation potential under the influence of various media supplemented with growth factors. Yet, most cell cultures include the use of animal sera, which raises safety concerns and might lead to variances in results. Electrospun nanoscaffolds represent suitable matrices for tissue engineering of skeletal muscle, combining both biocompatibility and stability.

We therefore aimed to develop a serum-free myogenic differentiation medium for the co-culture of primary myoblasts (Mb) and mesenchymal stromal cells derived from the bone marrow (BMSC) and adipose tissue (ADSC) on electrospun poly-ε-caprolacton (PCL)-collagen I-nanofibers.

**Results:**

Rat Mb were co-cultured with rat BMSC (BMSC/Mb) or ADSC (ADSC/Mb) two-dimensionally (2D) as monolayers or three-dimensionally (3D) on aligned PCL-collagen I-nanofibers. Differentiation media contained either AIM V, AIM V and Ultroser® G, DMEM/Ham’s F12 and Ultroser® G, or donor horse serum (DHS) as a conventional differentiation medium. In 2D co-culture groups, highest upregulation of myogenic markers could be induced by serum-free medium containing DMEM/Ham’s F12 and Ultroser® G (group 3) after 7 days. Alpha actinin skeletal muscle 2 (ACTN2) was upregulated 3.3-fold for ADSC/Mb and 1.7-fold for BMSC/Mb after myogenic induction by group 3 serum-free medium when compared to stimulation with DHS. Myogenin (MYOG) was upregulated 5.2-fold in ADSC/Mb and 2.1-fold in BMSC/Mb. On PCL-collagen I-nanoscaffolds, ADSC showed a higher cell viability compared to BMSC in co-culture with Mb. Myosin heavy chain 2, ACTN2, and MYOG as late myogenic markers, showed higher gene expression after long term stimulation with DHS compared to serum-free stimulation, especially in BMSC/Mb co-cultures. Immunocytochemical staining with myosin heavy chain verified the presence of a contractile apparatus under both serum free and standard differentiation conditions.

**Conclusions:**

In this study, we were able to myogenically differentiate mesenchymal stromal cells with myoblasts on PCL-collagen I-nanoscaffolds in a serum-free medium. Our results show that this setting can be used for skeletal muscle tissue engineering, applicable to future clinical applications since no xenogenous substances were used.

## Background

Volumetric muscle loss caused by trauma or aggressive tumor ablation is critical since it exceeds the natural regeneration capacity of skeletal muscle tissue. To reconstruct the resulting defects, donor tissue transfer, including free autologous muscle flaps, is considered as the current gold standard. This comes along with substantial donor site morbidity [1, 2]. Three-dimensional (3D) skeletal muscle tissue constructs created via tissue engineering hold promise for treating such volumetric defects, without sacrificing a complete autologous muscle at a certain donor site [3–6].

Engineering of skeletal muscle tissue requires easily expandable cells. Current studies have investigated the co-culture of bone-marrow derived mesenchymal stromal cells (BMSC) and primary myoblasts (Mb) [7, 8]. Unlike Mb, mesenchymal stromal cells (MSC) can be expanded widely without losing their differentiation ability. MSC are known to secrete several growth factors involved in the muscle regeneration process and to stimulate myoblast migration, proliferation, and cell survival [9].

Adipose derived stromal cells (ADSC) have gained popularity in the field of regenerative medicine because of several advantages associated with this cell type. ADSC can be obtained in abundant quantities and by minimally invasive procedures [10]. These cells can differentiate spontaneously into skeletal myoblasts, expressing myogenic markers and forming multinucleated myotubes when co-cultured with myogenic cells [11]. Tissue-engineered muscle constructs incorporating ADSC have displayed skeletal muscle regeneration potential [12].

Differentiation of Mb is typically induced by serum deprivation by switching from 10% fetal calf serum (FCS) to 2% donor horse serum (DHS) [13, 14]. Yet, serum components are often considered to be inconsistent and uncharacterized, which might lead to heterogenous results due to variations in different serum lots [15]. Sera are often used from different species, which raises safety concerns. Therefore, studies have focused on the use of serum-free media [13]. Although primary cultures are more suitable for investigating in vivo skeletal muscle growth and differentiation as they represent a model that is closer to in vivo situations and clinical applications, serum-free media have only been investigated on cell lines like C2C12 in terms of myogenic differentiation [16, 17]. Yet, this is inconvenient since cell lines are inappropriate for clinical applications.

To mimic axially oriented skeletal muscle tissue, it is necessary to cultivate cells on a proper 3D matrix. For inducing multinucleated myotubes, highly aligned nanofibers generated via electrospinning seem to be the most promising scaffold [18, 19]. Previous studies showed that pure collagen I nanofibers yielded excellent cellular affinity and myogenic differentiation of primary myoblasts seeded onto those fibers. They represented a nearly ideal scaffold for skeletal muscle tissue engineering purposes because of their similarity to the extracellular matrix. Unfortunately, they possess poor mechanical properties, leading to fast degradation and instability [20]. Composite nanofibers composed of both collagen I and the synthetic poly-ε-caprolactone (PCL) could overcome the obstacles stated above [21, 22].

In order to engineer transplantable skeletal muscle tissue for future translational applications, we aimed to establish a serum-free medium for myogenic differentiation of primary Mb and MSC on PCL-collagen I-nanofiber scaffolds.

## Results

### Myoblast and ADSC characterization

**Fig. 1:**
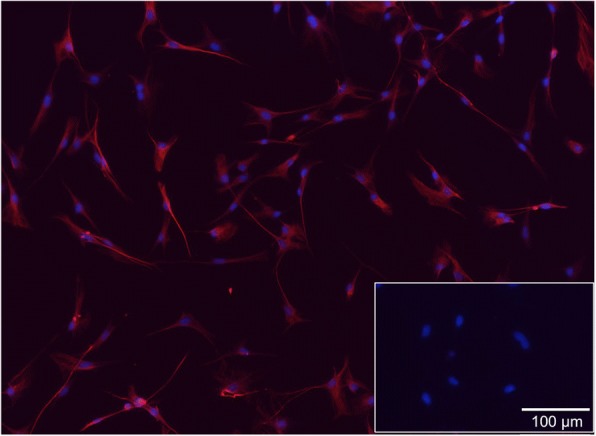
Fluorescence microscopy of desmin-positive rat primary myoblasts. Myoblasts were enriched with a preplate-technique by seeding the supernatant of isolated cells into new flasks after two, 24, and 48 h. The third preplate was further passaged until passage 3. Merge of DAPI (blue) and desmin (red, with Alexa Fluor 647 as secondary antibody) showed that nearly all cells were myoblasts (desmin-positive). Insert shows fibroblasts isolated from rat skin negative for desmin

**Fig. 2:**
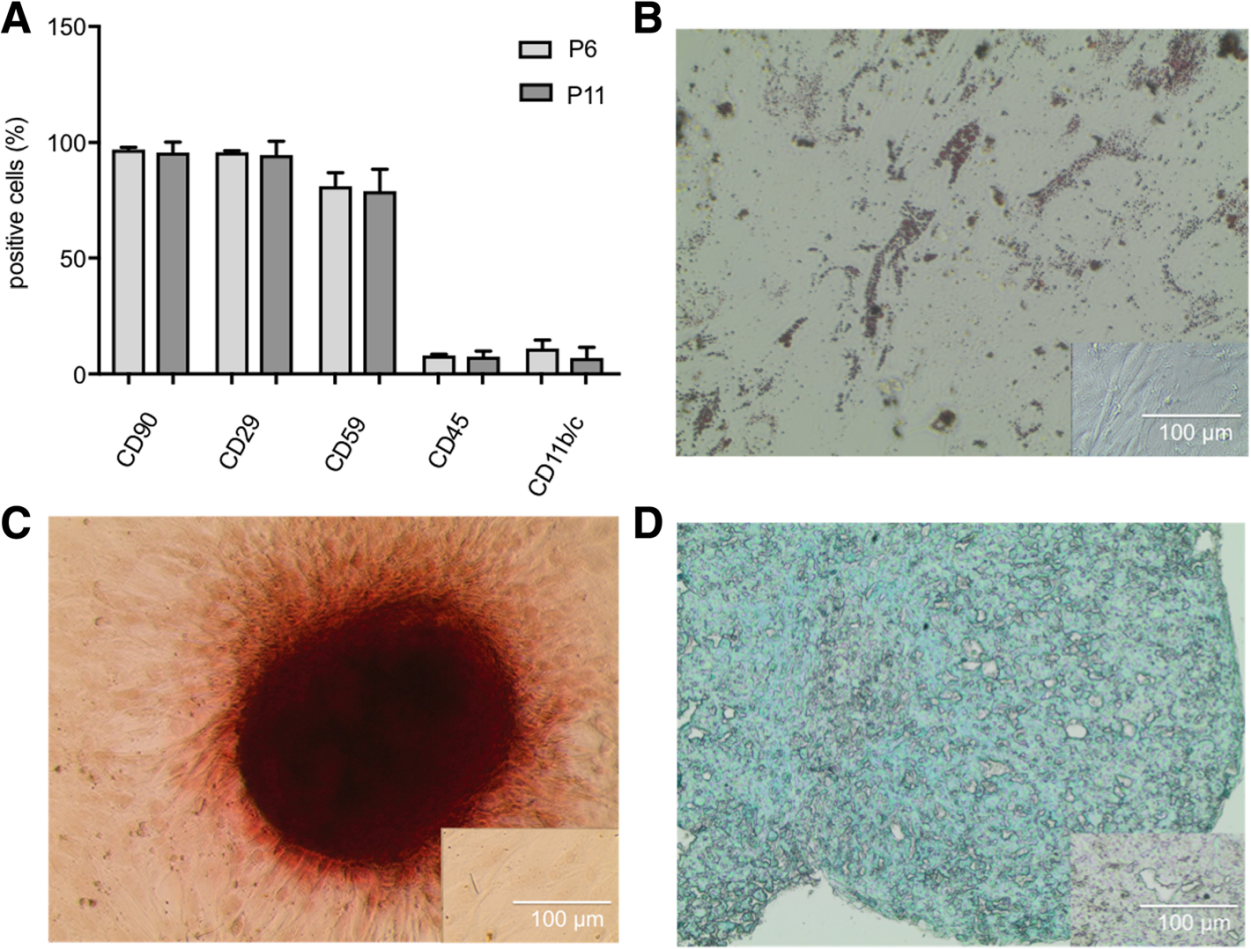
Characterization of adipose derived stem cells (ADSC). **a** ADSC were analyzed for cell surface markers in passage 6 (P6) and passage 11 (P11). Over 90% of cells were positive for CD90 and CD29 and negative for CD45 and CD11b/c in both passages. Paired t-test between P6 and P11 showed no differences in expression of surface markers. ADSC in passage 4 were differentiated into adipocytes (**b**), osteocytes (**c**), and chondrocytes (**d**). Lipid vacuoles were visualized with oil red O staining (**b**), calcium deposits were stained with Alizarin Red S (**c**), and proteoglycans of chondrogenic pellets were detected by Alcian blue staining (**d**). Inserts represent ADSC, cultured in proliferation medium as negative controls

Mb from passage 3 and pre-plate 3 showed a > 95% positive staining for the muscle-specific marker desmin (Fig. 1). ADSC were successfully differentiated into chondrogenic, osteogenic, and adipogenic lineage (Fig. 2b-d). Furthermore, they were analyzed for cell surface markers in low (P6) and high passages (P11). Over 90% of cells were positive for CD90 (96.9 ± 0.98% and 95.67 ± 4.57%, respectively, *n* = 3) and CD29 (95.8 ± 0.66% and 94.6 ± 5.98%, respectively, n = 3) and negative for CD45 (8.08 ± 0.51% and 7.5 ± 2.44%, respectively, *n* = 3) and CD11b/c (11.02 ± 3.63% and 6.95 ± 4.56%, respectively, *n* = 3) in both passages (Fig. 2a).

### The effect of serum-free media on 2D mono-cultures of Mb and co-cultures of BMSC/Mb and ADSC/Mb

Mb were mono- and co-cultured with BMSC (BMSC/Mb) and ADSC (ADSC/Mb) as monolayers in differentiation media for different time periods.

**Fig. 3:**
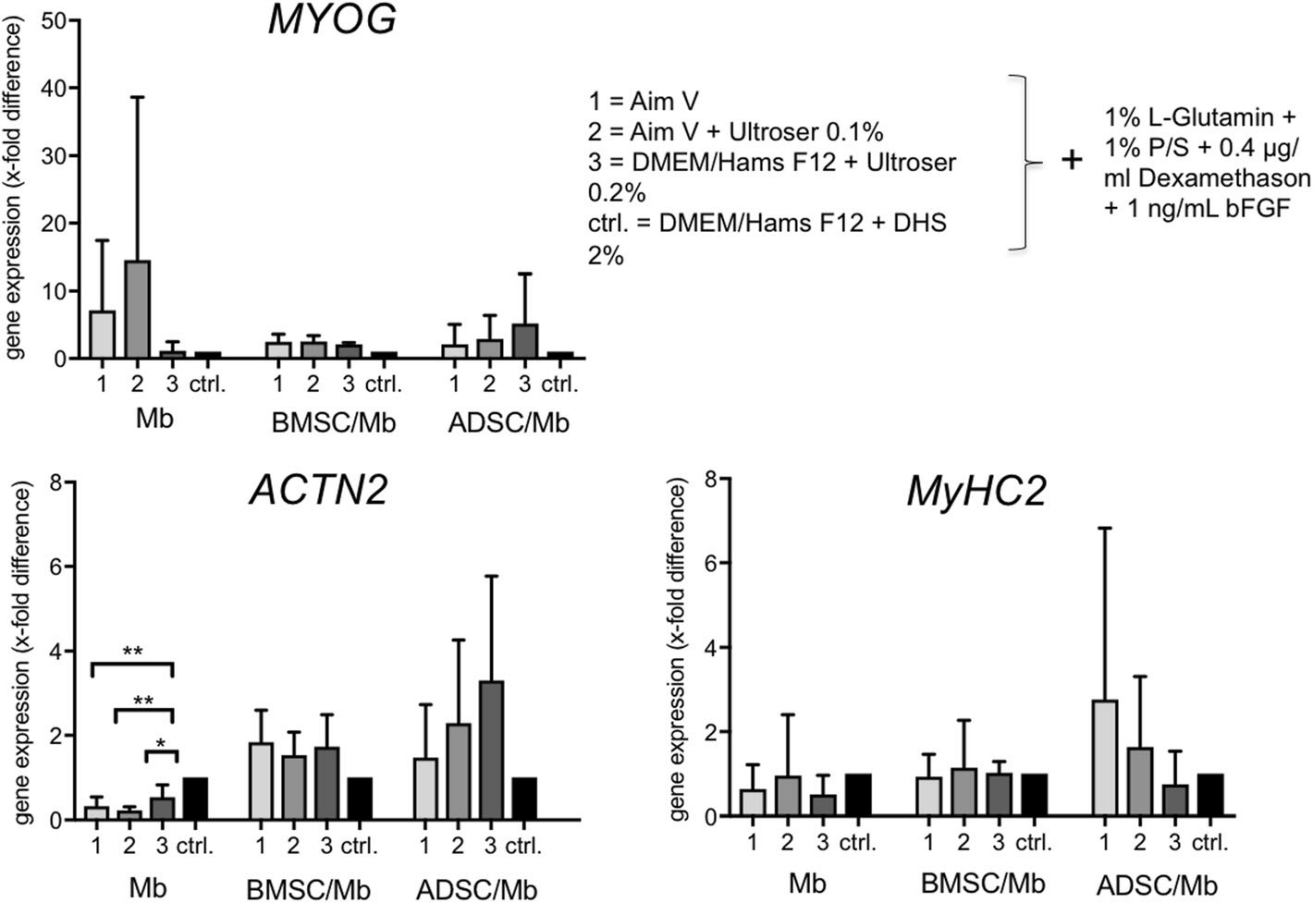
Gene expression of myogenic markers in Mb, BMSC/Mb, and ADSC/Mb after serum-free myogenic differentiation. Expressions are demonstrated in x-fold difference compared with Mb, BMSC/Mb, ADSC/Mb stimulated with standard myogenic differentiation medium (ctrl. = control = 1) using the 2^-ΔΔCt^-method. Markers are presented as mean ± standard deviation. In Mb, serum-free differentiation led to a downregulation of *ACTN2* (alpha actinin skeletal muscle 2). Statistical differences were tested with one-way ANOVA and Bonferroni‘s correction for multiple comparisons (*n* = 3). Levels of significance were * *p* ≤ 0.05, ** *p* ≤ 0.01

After 7 days, gene expression of different myogenic markers could be observed under all conditions (Fig. 3). In Mb, expression of *ACTN2* (alpha actinin skeletal muscle 2) and *MyHC2* (myosin heavy chain 2) was lower under serum-free differentiation. *ACTN2* was significantly downregulated after stimulation with all groups of serum-free media compared to stimulation with differentiation medium containing DHS (*p* = 0.0042). On the contrary, *ACTN2* and *MyHC2* were both upregulated in co-culture groups. This was most noticeable in ADSC/Mb, though differences were not statistically significant. Group 3 led to the highest upregulation of *ACTN2* and *MYOG* (myogenin) in ADSC/Mb. In Mb, group 1 and 2 led to an upregulation of *MYOG*. In BMSC/Mb, *ACTN2*, *MyHC2*, and *MYOG* were expressed relatively similar throughout all groups.

**Fig. 4:**
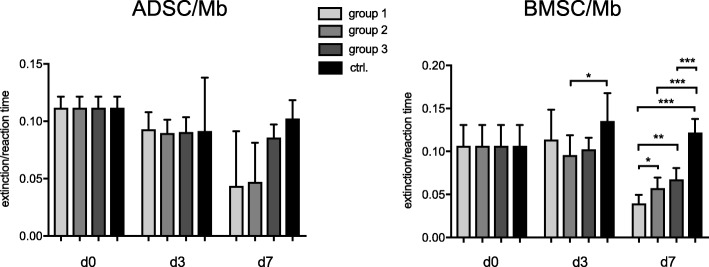
CK activity of ADSC/Mb and BMSC/Mb after (serum-free) myogenic differentiation. CK activity was measured as extinction during minute 2–6 of reaction time. Cells were allowed to differentiate for three (d3) and 7 days (d7) in serum-free media group 1, 2, 3, and standard myogenic differentiation medium (ctrl.) after 2 days of proliferation (d0). Values are presented as mean ± standard deviation. In BMSC/Mb, ctrl. Led to higher CK activity compared to all serum-free media. Highest CK activity was seen for group 3 of all serum-free media after 7 days of myogenic differentiation. Statistical differences were tested with one-way ANOVA and Bonferroni’s correction for multiple comparisons (*n* = 3). Levels of significance were * p ≤ 0.05, ** p ≤ 0.01, *** *p* ≤ 0.001

Creatine kinase (CK) activity showed a decrease in ADSC/Mb after 3 days of myogenic differentiation irrespective of the differentiation medium (Fig. 4). After 7 days of differentiation, CK activity further decreased in group 1 and 2 while it was stable for group 3 and slightly increased after stimulation with standard differentiation medium. Differences between groups were not statistically significant. For BMSC/Mb, results were similar with a slight decrease of CK activity in group 2 and 3 after 3 days of differentiation and a significant increase of CK activity after standard differentiation compared to group 2 (*p* = 0.048). After 7 days, CK activity decreased for all groups, with the strongest decrease in group 1, followed by group 2, 3, and the control group. One-way ANOVA and Bonferroni’s correction for multiple comparisons showed significant differences between all groups with highly significant differences between the control group and all serum-free groups (*p* < 0.001).

### Cell viability on PCL-collagen I-nanoscaffolds

**Fig. 5:**
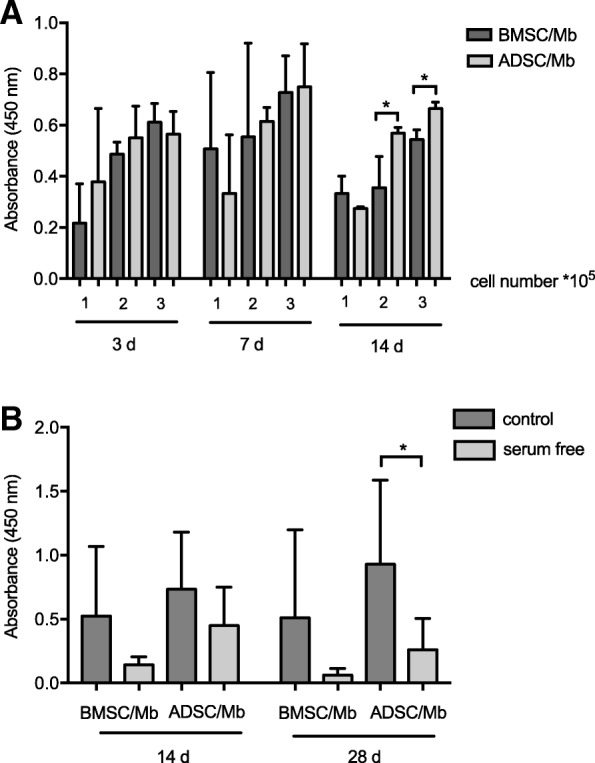
Cell viability on PCL-collagen I-nanoscaffolds. **a** BMSC/Mb and ADSC/Mb were seeded at different densities (1 × 10^5^, 2 × 10^5^, 3 × 10^5^) on PCL-collagen I-nanoscaffolds and were allowed to proliferate for different time periods: 3 days (d), 7 d, or 14 d. Cell viability was determined by WST 8-assay. Absorbance at a wave length of 450 nm is expressed as mean ± standard deviation. At 14 days, ADSC showed a significantly higher cell viability for 2 × 10^5^ and 3 × 10^5^ cells in comparison to BMSC/Mb. **b** BMSC/Mb or ADSC/Mb were seeded at a density of 3 × 10^5^ cells on PCL-collagen I-nanoscaffolds. Myogenic differentiation was induced by standard differentiation medium (control) or group 3 serum-free medium. WST 8-assay was repeated after 14 and 28 days of differentiation. Results showed decreased cell viability after serum free differentiation. ADSC/Mb showed higher cell viability compared to BMSC/Mb for all groups. Statistical differences were tested with repeated measures ANOVA for comparison between paired variables and Tukey’s multiple comparisons test as posthoc test at different time points. Pairwise comparison between BMSC/Mb and ADSC/Mb was done using unpaired t-test (n = 3). Level of significance was * *p* ≤ 0.05

Cell viability was assessed via WST-8-assay as absorbance at 450 nm. At all time points, an increasing cell viability was noted with relation to the number of seeded cells (Fig. 5a). A trend towards an increasing cell viability was seen at 7 days for all groups, with 3 × 10^5^ having the highest mean cell viability for both BMSC/Mb as well as ADSC/Mb. Although pairwise comparison showed no significant difference, ADSC/Mb had a higher mean cell viability than BMSC/Mb. At 14 days, ADSC/Mb showed a significantly higher cell viability for 2 × 10^5^ and 3 × 10^5^ cells in comparison to BMSC/Mb (*p* = 0.041 and *p* = 0.011, respectively). For further experiments involving myogenic differentiation of ADSC/Mb and BMSC/Mb on scaffolds, a total cell number of 3 × 10^5^ as well as a proliferation time of 7 days were chosen due to above stated results.

WST-8-assay was repeated after 14 and 28 days of myogenic differentiation, both with and without serum (group 3) (Fig. 5b). Results showed decreased cell viability after serum free differentiation at 14 days and a further decrease at 28 days, which was significant for ADSC/Mb (p = 0.041). A higher viability could be shown for ADSC/Mb compared to BMSC/Mb in all groups, though differences were not significant.

### Myogenic differentiation on PCL-collagen I-nanoscaffolds

**Fig. 6:**
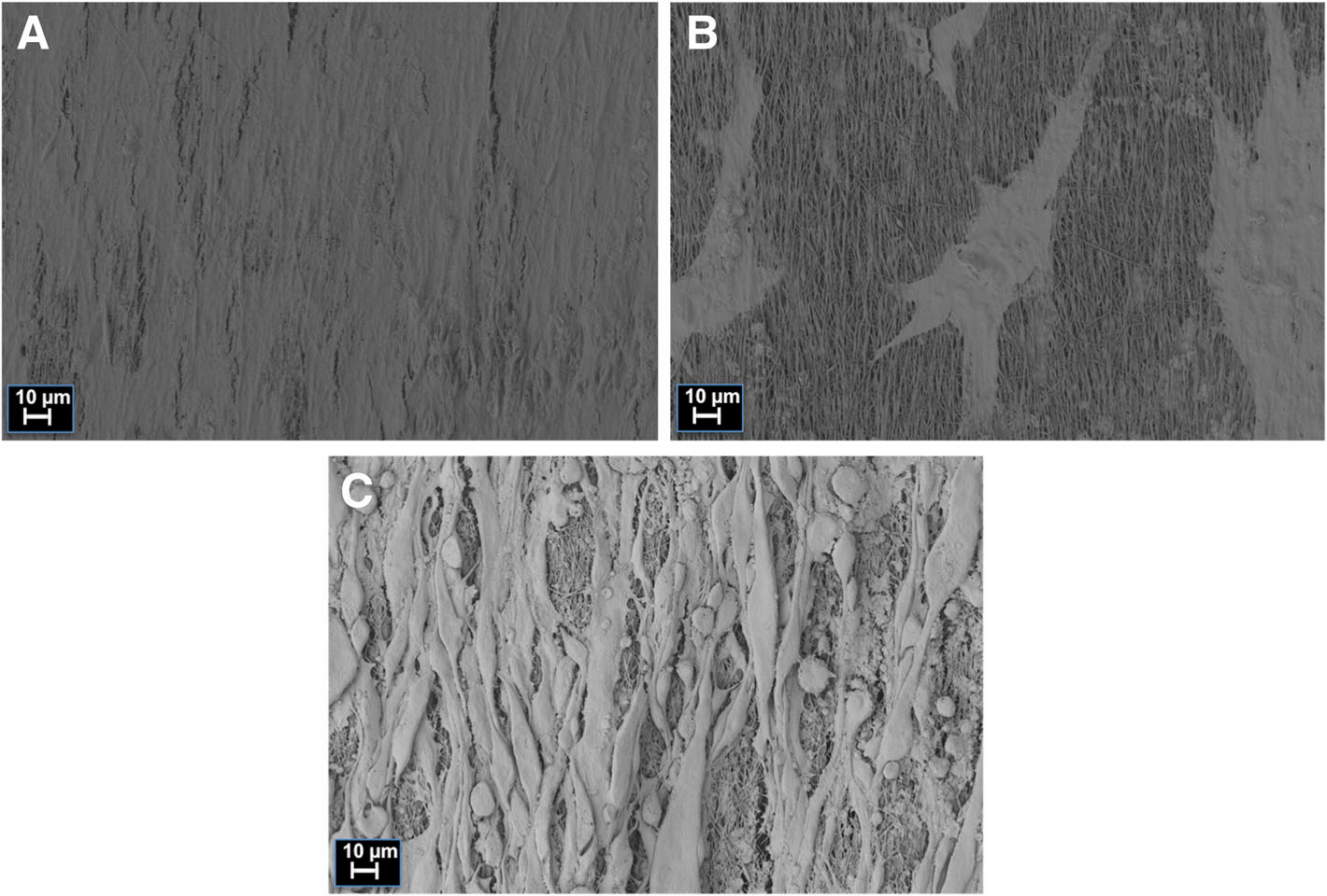
Scanning electron microscopy of ADSC/Mb, BMSC/Mb, and C2C12 on PCL-collagen I-nanoscaffolds after long-term myogenic differentiation. After 28 days of myogenic differentiation, (**a**) ADSC/Mb were confluent, covering almost the entire surface of the scaffold. **b** BMSC/Mb were less densely spread on the scaffold. **c** C2C12 cells showed parallel alignment on the scaffolds with myotube-like structures

With SEM (scanning electron microscopy) images, configuration of attached cells on PCL-collagen I-nanoscaffolds could be analyzed (Fig. 6). After 28 days of myogenic differentiation, C2C12 cells showed parallel alignment on the scaffolds with myotube-like structures. ADSC/Mb were confluent, covering almost the entire surface. BMSC/Mb were less densely spread on the scaffold.

**Fig. 7:**
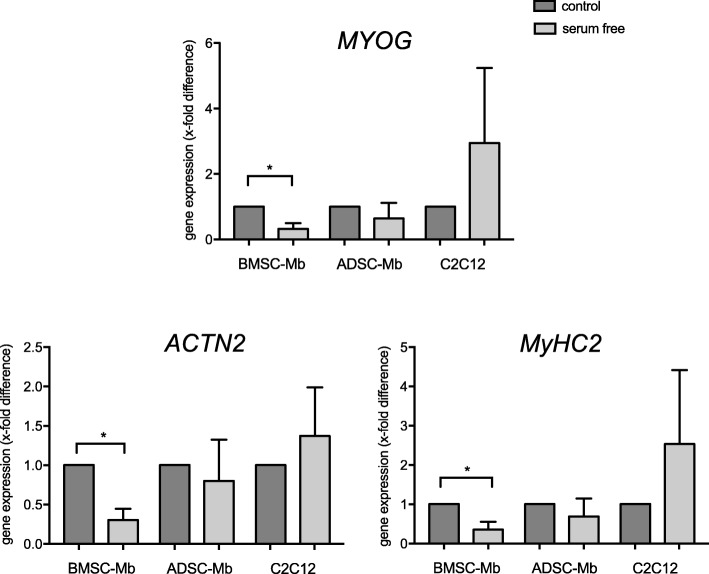
Myogenic differentiation of BMSC/Mb, ADSC/Mb, and C2C12 after long-term stimulation on PCL-collagen I-nanoscaffolds. Cells were stimulated with group 3 serum-free medium. Expressions are demonstrated in x-fold difference compared with BMSC/Mb and ADSC/Mb, stimulated with standard myogenic differentiation medium (control = 1) using the 2^-ΔΔCt^-method. Markers are presented as mean ± standard deviation. *MyHC2* (myosine heavy chain 2), *ACTN2* (alpha actinin skeletal muscle 2), and *MYOG* (myogenin) were downregulated after 28 days of serum free myogenic differentiation for BMSC/Mb compared to controls. Statistical differences were tested with paired t-test or Wilcoxon test, as appropriate (*n* = 3). Level of significance was * *p* ≤ 0.05

2D mono- and co-culture-groups showed rather heterogenous results in terms of gene expression of key myogenic markers. But since CK assay showed higher CK activity for both ADSC/Mb and BMSC/Mb stimulated with group 3 compared to other serum-free groups, group 3 serum-free medium was chosen to be further analyzed for 3D culture on PCL-collagen I-nanoscaffolds. Gene expression of *MyHC2*, *ACTN2*, and *MYOG* (Fig. 7) was downregulated after 28 days of serum-free myogenic differentiation for BMSC/Mb compared to controls (*p* = 0.0313, *p* = 0.0137, *p* = 0.02, respectively). C2C12, however, showed higher gene expression of *MyHC2* (2.54-fold ±1.86-fold), *ACTN2* (1.38-fold ±0.62-fold), and *MYOG* (2.95-fold ±2.30-fold) after serum free differentiation over the same time period, although differences were not statistically significant. For ADSC/Mb a slight trend in favor of the control group was detected.

**Fig. 8:**
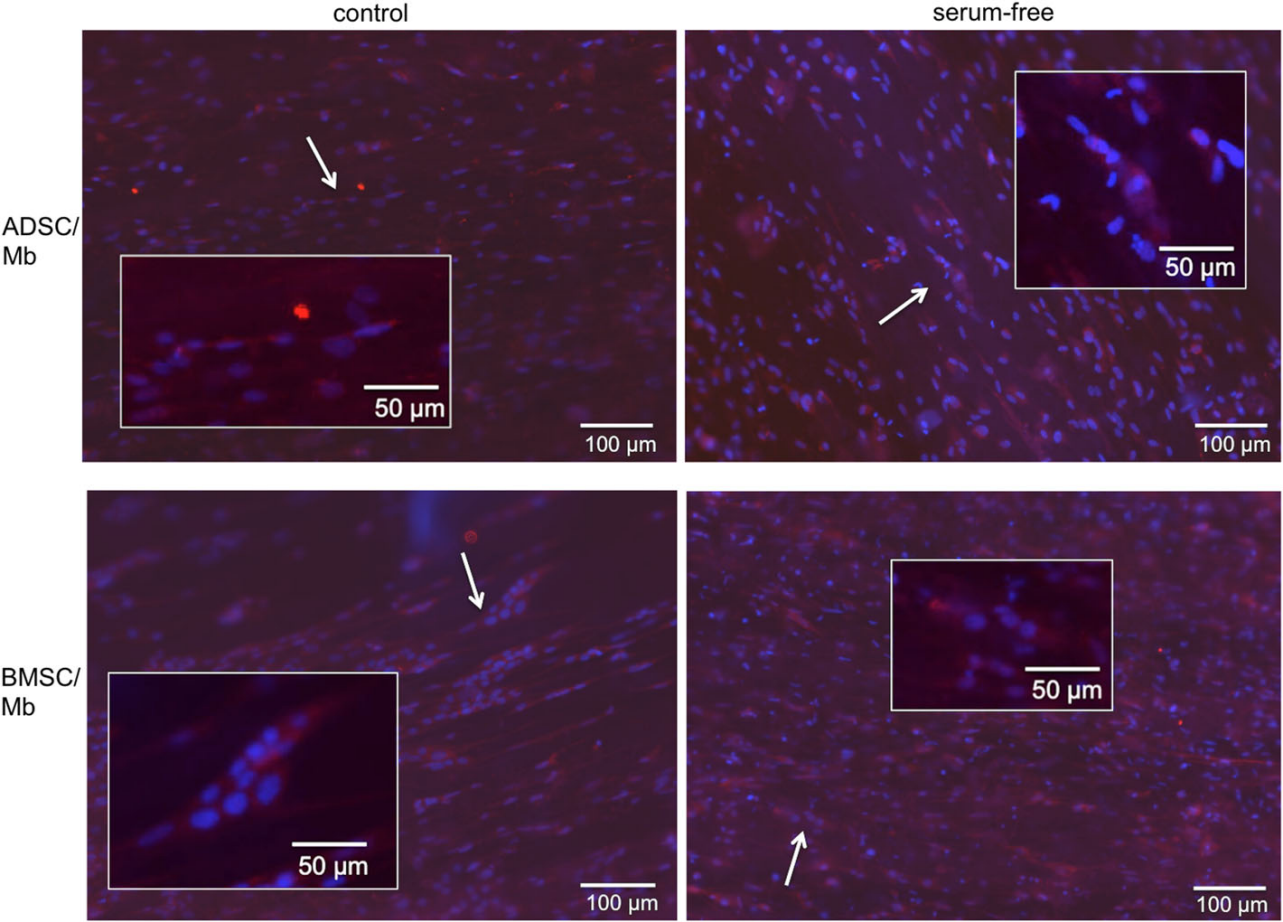
MHC expression after long-term myogenic differentiation on PCL-collagen I-nanoscaffolds. With fluorescence microscopy, the myogenic differentiation potential of BMSC/Mb and ADSC/Mb seeded on PCL-collagen I-nanoscaffolds was analyzed after 28 days of standard and group 3 serum-free myogenic differentiation. Both control and serum-free media led to positive expression of myosin heavy chain for BMSC/Mb and ADSC/Mb. Furthermore, multinucleated cells as possible myotube formation were found (representatives are marked with arrows and magnified in inserts)

With fluorescence microscopy, the myogenic differentiation potential of BMSC/Mb and ADSC/Mb seeded on PCL-collagen-nanoscaffolds was analyzed after 28 days of standard and serum free myogenic differentiation. Both control and group 3 serum-free media led to positive expression of myosin heavy chain for BMSC/Mb and ADSC/Mb. Furthermore, multinucleated cells as possible myotube formation were found (Fig. 8).

## Discussion

This is, to the best of our knowledge, the first report of a serum-free medium for myogenic differentiation of primary MSC in co-culture with Mb on a biocompatible matrix.

Several studies have reported the influence of differentiation medium without serum on cell lines like C2C12 or L6 [13], but the influence on co-cultures of primary Mb with MSC on PCL-collagen I-nanoscaffolds has not been investigated so far. The choice of serum-free media in the present study was based on promising media reported in the literature for 2D as well as 3D C2C12-culture [16, 17].

Surprisingly, CK activity decreased after induction of myogenic differentiation, although it is known to correlate with myotube formation. This could have been evoked by completely changing the growth medium into (serum free) differentiation medium. Others have suggested that a gradual decline in growth factors should rather be employed to enable cell survival and maturation into myotubes [23, 24]. Another explanation could be myotube formation with detachment from plastic surfaces in this 2D setting.

Electrospun PCL-collagen I-nanoscaffolds represent a biocompatible and stable matrix for 3D tissue engineering of skeletal muscle. Recently, we were able to electrospin those nanofibers, using acetic acid as a benign solvent, facilitating translational research [21]. The matrices enabled long-term differentiation of skeletal progenitor cells and parallel alignment on the nanofibers. While 2D co-cultures of BMSC/Mb and ADSC/Mb showed no expression of myosin heavy chain after 7 days (data not shown), cells expressed the myosin motor protein when seeded on the scaffolds and allowed to differentiate for 28 days. Others have described a similar time period for differentiating MSC to express key myogenic markers, especially myosin heavy chain as a late myogenic marker [25–28]. Multinucleated cells, indicating possible myotube formation, could be shown under both standard and serum free differentiation conditions. This condition also led to positive gene expressions of key myogenic markers. While BMSC/Mb showed lower expression of all myogenic markers under serum free conditions, these markers were upregulated in C2C12 cells up to 2.95-fold under stimulation with DMEM/Ham’s F12 and Ultroser® G compared to stimulation with standard myogenic differentiation medium, containing DHS. This is in accordance to other studies [16, 17]. Ultroser® G contains growth factors like epidermal growth factor, transforming growth factor, insulin-like growth factor (IGF), fibroblast growth factor, insulin, thyroxine, and dexamethasone, which are also present in animal serum and are known to enhance myogenic differentiation [16, 17]. It has been shown to enhance myogenic differentiation in 3D cultures of C2C12, even more than IGF [17]. More recent studies have described the utilization of the neuronal cell culture serum-free supplement B27 for myogenic differentiation of myogenic progenitors derived from human embryonic and induced pluripotent stem cells (IPS) [29]. However, the clinical application of embryonic or IPS cells might raise ethical and safety concerns. The effect of serumfree media on myogenic differentiation seems to depend on the stimulated myoblast types since the immortalized mouse cell line C2C12 has shown to be enhanced in myotube formation through serum free media compared to other myoblast cells [13]. This is in accordance to the results in our study, showing that C2C12 have higher expression of MYOG, ACTN2, and MHC2 after serum-free differentiation, while corresponding gene expression in co-cultures of MSC and Mb were reduced. Myogenic differentiation of MSC necessitates growth factors and direct cell contact with co-cultured myoblasts [7, 11, 30]. It might be possible that Ultroser® G lacks some growth factors contained in DHS, which are crucial for myogenic differentiation of primary cells. A recent study by Sassoli et al. showed that platelet rich plasma (PRP) as a serum substitute in combination with BMSC positively influenced proliferation and differentiation of myoblasts in vitro. However, similar to animal sera, PRP’s limitation is its heterogeneity due to non-standardized preparation procedures [31]. Future studies might investigate other growth factors promoting myogenic differentiation. In our study, ADSC/Mb were able to myogenically differentiate under both serum-free and standard differentiation conditions, whereas BMSC/Mb showed significant downregulation of myogenic markers after serum free differentiation. A different growth factor secretion profile could be a possible reason for this [10, 32]. Our results are in accordance with a study published by Stern-Straeter et al., who were able to detect gene expression of myogenic markers in human ADSC after serum-free differentiation, but not in BMSC. In their study, different MSC media, unconditioned or conditioned with the supernatant of human satellite cells, were used [26].

There are several limitations of this study. First, high standard deviations as well as small sample size, sometimes led to differences that were not statistically significant. But due to time-consuming production of PCL-collagen I-nanoscaffolds and isolation of cells, it was not possible to retrieve groups of larger sample size. Furthermore, maintaining and myogenic differentiation of primary cells isolated from adult muscle tissue is a greater challenge than culturing cell lines like C2C12, which is used in a great number of other studies [13, 16, 18, 19, 33]. Since those cells do not display any contact inhibition, it is difficult to compare C2C12 to primary cells. Thus, results of ADSC/Mb and BMSC/Mb should be evaluated independently of C2C12, which should rather serve as positive control. In the past, we were able to overcome the restricted proliferation capacity of Mb by co-culturing them with BMSC [7, 8]. In the present study, we successfully isolated ADSC and proved their mesenchymal stem cell characteristics to be present in high passages up to passage 11. This means that a small number of cells can be largely expanded, making this kind of cell attractive for regenerative medicine, especially in co-culture with primary myoblasts. Second, we did not distinguish between living and dead cells in our 3D setting. A live-dead assay can help to evaluate cell cytotoxicity of the scaffolds as well as clarify cell status, a potential confounding factor for myogenic differentiation. However, WST-8 assay is a commonly used tool for cytotoxicity testing since it is sensitive and reproducible [34]. The amount of formazan dye generated is directly proportional to the number of living cells and several groups have used this assay for evaluating new biomaterials or tissue engineered constructs [34–36]. WST-8-assay showed higher viability for ADSC compared to BMSC, indicating higher proliferation capacity, which has also been shown by others [37]. Third, using different culture settings (2D vs 3D), we were not able to analyze expression of myogenic markers over the course of time. But, as already mentioned above, monolayered cells did not survive for a time period longer than 7 days, probably due to abrupt serum deprivation. Given the results after long term differentiation, we suppose that PCL-collagen I-nanoscaffolds serve as a platform, promoting cell survival, adhesion, and myogenic differentiation.

## Conclusion

The co-culture of ADSC and BMSC with Mb necessitates PCL-collagen I-nanoscaffolds as an adequate 3D matrix for long-term myogenic differentiation. Serum-free differentiation is feasible, particularly for ADSC/Mb. This biocompatible model for skeletal tissue engineering can be transferred to further in vivo and translational research and brought us one step closer to future clinical applications.

## Methods

### Myoblast cell culture

20-week old male Lewis rats (Chales River, Wilmington, Massachusetts, USA) were euthanized by cardiac exsanguination under isoflurane inhalation anaesthesia. Mb were isolated from hind limb muscle of male Lewis rats as described previously [8]. A preplate-technique was used for enrichment of Mb [38]. Briefly, cells were plated into type I collagen-coated flasks (rat tail collagen, Sigma Aldrich, St. Louis, Missouri, USA). For cell culture, Ham’s F10 medium (Gibco, Carlsbad, California, USA) containing 25% FCS (Biochrom GmbH, Berlin, Germany), 1.25% penicillin/streptomycin (P/S) (Biochrom GmbH) and 2.5 ng/ml basic fibroblast growth factor (bFGF) (Peprotech, Hamburg, Germany) was used. After 2 hours, the supernatant containing non-adherent cells was collected and replated in a new coated flask. This step was repeated every 24 h. The third preplated cells were further passaged. Medium was changed every other day. Mb of passage 3 were used for all experiments. Desmin immunofluorescence (ab8470, Abcam, Cambridge, UK) showed a ratio of approximately 95% myoblasts (Fig. 1). Fibroblasts isolated from rat skin via dispase (Sigma Aldrich, St. Louis, Missouri, USA) and collagenase Type II (Biochrom GmbH) served as negative control.

### BMSC and ADSC cell culture, characterization and differentiation

Rat BMSC were isolated from the bone marrow of male Lewis rats and characterized as described previously [39, 40]. ADSC were enzymatically isolated from the inguinal subcutaneous fat tissue of male Lewis rats. Briefly, the adipose tissue was minced and digested in 0.075% collagenase Type I (Biochrom GmbH) and incubated at 5% CO2 and 37 °C for 1 h under continuous agitation. The cell suspension was filtered through a 100 μm nylon cell strainer (MACS® SmartStrainer, Miltenyi Biotec GmbH, Bergisch Gladbach, Germany) to remove cellular debris. After centrifugation and lysis of red blood cells the cell suspension was again filtered through a 70 μm nylon cell strainer (MACS® SmartStrainer, Miltenyi Biotec GmbH). The cells were resuspended in growth medium containing DMEM Ham’s F12, 10% FCS, 1% L-Glutamin, 1% P/S (all from Biochrom GmbH). After 24 h, the cells were washed to remove non-adherent cells. Phenotype was assessed by the cells’ ability to differentiate into chondrocytes, adipocytes and osteocytes with specific differentiation media (Pelobiotech GmbH, Planegg, Germany). Flow cytometry was performed on cells from the 6th and 11th passage to evaluate the cell population. Cells were incubated with the following fluorochrome-conjugated antibodies: CD90, CD29, CD59, CD45, CD11b/c (Miltenyi Biotec GmbH). Detection of fluorochrome labeling was performed on a fluorescence activated cell sorting cytometer (FACSCalibur) with BD CellQuest™ software (BD Bioscience, Franklin Lakes, NJ, USA) and analyzed with FlowJo® software (Tree Star, Ashland, OR, USA). ADSC and BMSC of passage 11 were used for experiments.

### Differentiation conditions

Mb monocultures, co-cultures of BMSC/Mb and ADSC/Mb were differentiated as monolayers for 7 days. Differentiation media contained 1% L-Glutamin, 1% P/S, 0,4 μg/ml dexamethasone (Sigma Aldrich), 1 ng/ml bFGF either supplemented with DMEM/Ham’s F12 + 2% DHS (Biochrom GmbH) (standard serum-containing differentiation medium), AIM V (serum-free medium, Thermo Fisher Scientific Inc., Waltham, MA, USA, group 1), AIM V + 0.1% Ultroser® G (Cytogen GmbH, Wetzlar, Germany) (group 2), or DMEM/Ham’s F12 + 0.2% Ultroser® G (group 3) (Table 1).

**Table 1:** Myogenic differentiation media

Group	Contains 1% L-Glutamin, 1% P/S, 0.4 μg/ml Dexamethason, 1 ng/mL bFGF +
1	AIM V
2	AIM V + 0.1% Ultroser
3	DMEM/Ham’s F12 + 0.2% Ultroser
standard (ctrl.)	DMEM/Ham’s F12 + 2% DHS

For co-culture experiments, Mb/BMSC or Mb/ADSC were seeded in a ratio of 1:1 in 6-well culture plates at a density of 3 × 10^5^ cells in expansion medium (DMEM/Ham’s F 12, 10% FCS, 1% L-Glutamin, 1% P/S). After 48 h, medium was replaced by differentiation medium. For 3D cultivation, BMSC/Mb or ADSC/Mb were seeded in a ratio of 1:1 at a density of 3 × 10^5^ cells on PCL-collagen I-nanoscaffolds and allowed to proliferate for 7 days before differentiation was induced by group 3 serum-free medium or control medium. Medium was changed every other day. For each experiment, Mb from three different isolations were used.

### Electrospinning of PCL-collagen I-nanofibers and cell seeding

PCL-collagen I-nanofibers were produced by electrospinning as described previously [21]. Briefly, PCL (Sigma Aldrich) was blended with bovine collagen type I (Symatese, Lyon, France) in a ratio of 2:1 at a 10-wt.%solution, using 90% acetic acid (Carl Roth GmbH, Karlsruhe, Germany) as a solvent. Electrospinning was performed on a standard electrospinning machine and parallel nanofibers were spun onto parallel metal rods on a custom made rotating drum. The aligned fibers were collected on plastic rings with 10 mm diameter (Minusheet carrier, Minucells and Minutissue Vertriebs GmbH, Bad Abbach, Germany). The area of the resulting scaffolds measured approximately 0,8 cm^2^. Scaffolds were sterilized in 70% ethanol, washed with PBS afterwards and placed into 24 well-plates while they were soaked in DMEM/Ham’s F12 for approximately 1 h at 37 °C. BMSC/Mb or ADSC/Mb were seeded with 100 μL thickened medium containing expansion medium and dissolved methyl cellulose (Sigma Aldrich) on PCL-collagen I-nanoscaffolds at three different densities: 1 × 10^5^ cells, 2 × 10^5^ cells, 3 × 10^5^ cells (in a ratio of 1:1, repectively). Each group was cultured in expansion medium for three, seven, and 14 days. After each time period, WST-8-assay (Promokine, Promocell GmbH, Heidelberg, Germany) of the seeded scaffolds was performed by adding 50 μL of Colorimetric Cell Viability Kit I-solution onto each scaffold. After 2 h of incubation, absorbance was measured at 450 nm with Photometer Thermo Scientific™ Multiskan™ GO to assess cell viability. For the following experiments a total cell number of 3 × 10^5^ cells was used with a proliferation period of 7 days prior to induction of differentiation. Differentiation was induced over a period of 28 days. WST-8-assay was repeated after 14 and 28 days of differentiation for evaluation of cell viability.

### RNA isolation and quantitative PCR analysis

In 2D and 3D mono- and co-cultures the gene expression rate of *MyHC2, ACTN2*, and *MYOG* was analyzed. As housekeeping gene, *RPL13a* (ribosomal protein L13a) was used. RNA of the samples was extracted using the RNeasy micro kit (Qiagen GmbH, Hilden, Germany) according to the manufacturer’s protocols. RNA was reverse-transcribed into cDNA using a QuantiTect Reverse Transcription Kit and a Sensiscript Reverse Transcription Kit (both from Qiagen GmbH). cDNA was amplified through quantitative real-time PCR using SsoAdvanced Universal SYBR Green PCR Supermix (Bio-Rad, Hercules, CA, USA) and Light Cycler (Bio-Rad CFX96 Touch™). Evaluation of gene expression was performed using the 2^-ΔΔCt^ method. C2C12 cells (ATCC, Manassas, Virginia, USA) served as positive controls. The primer sequences used are given in Table 2.

**Table 2:** Primer sequences

	Forward primer	Reverse primer
*MYOG*	TGAGAGAGAAGGGAGGGAAC	ACAATACACAAAGCACTGGAA
*MyHC2*	TGACTTCTGGCAAAATGCAG	CCAAAGCGAGAGGAGTTGTC
*ACTN2*	TCACTGAGGCCCCTTTGAAC	AGACAGCACCGCCTGAATAG
*RPL13a*	CTCATGAGGTCGGGTGGAAG	AGAGCTGCTTCTTCTTCCGG

### Creatine kinase activity

BMSC/Mb or ADSC/Mb were seeded in a ratio of 1:1 at a density of 3 × 10^5 cells in 6-well plates as monolayers and allowed to proliferate for 2 days before differentiation was induced by standard and serum-free media. CK activity was colorimetrically determined (Abcam) after 2 days of proliferation, three and 7 days of differentiation. Cells were resuspended in 50 μL CK assay buffer and 5 μL of the suspension was used for reaction. The reaction is based on enzymatic conversion of creatine and adenosine triphosphate into phosphocreatine and adenosine diphosphate (ADP) by CK. ADP is subsequently applied to form nicotinamide adenine dinucleotide (NADH) after reaction mix is added to each sample. The amount of NADH generated by creatine kinase was determined photometrically at 450 nm with Thermo Scientific™ Multiskan™ GO during minute 2–6 of reaction time since after 6 min, the activity of the samples was found to have reached a plateau.

### Immunofluorescence

For freshly isolated Mb, third preplates in passage 3 were seeded on collagen-coated 48-well plates at a density of 5 × 10^3^ cells. After 24 h, cells were fixed with formaldehyde (Carl Roth GmbH) and washed and incubated in blocking buffer consisting of PBS with 1.5% FCS and 0.25% TritonX (Carl Roth GmbH) for 1 h at room temperature. After washing with TBS-T buffer (100 mM Tris and NaCl in distilled water, 1 ml Tween20 per 1 L, pH 7.6), cells were incubated with desmin primary antibody (ab8470, Abcam) at 0.5 μg/ml for 1 h.

BMSC/Mb or ADSC/Mb were seeded in expansion medium at a density of 3 × 10^5 cells on PCL-collagen I-nanoscaffolds. After 7 days, the medium was switched to differentiation medium with or without serum. After 4 weeks, scaffolds were fixed, washed, and blocked as described above. For staining, scaffolds were covered with anti-fast myosin skeletal heavy chain antibody (ab91506, Abcam) diluted 5 μg/ml in blocking buffer for 1 h at room temperature.

Alexa Fluor 647 goat anti-rabbit IgG H&L (ab150083, Abcam) was used as secondary antibody at 4 μg/ml for 30 min at room temperature for both Mb and co-cultures. Probes were counterstained with DAPI 1 μg/ml (diamidine-phenylindole-dihydrochloride, Thermofisher Scientific Inc.) for 5 min. Cells were subsequently analyzed and digitally photographed with a fluorescence microscope (IX83, cellSens, software, Olympus, Hamburg, Germany).

C2C12 and skeletal muscle slides served as positive control while fibroblasts isolated from rat skin served as negative control.

### Scanning electron microscopy

BMSC/Mb, ADSC/Mb, or C2C12 were seeded on PCL-collagen I-nanoscaffolds at a density of 3 × 10^5^ cells and allowed to proliferate for 7 days bevor differentiation was induced with standard differentiation medium for 28 days. Microstructural analysis of the seeded scaffolds was performed using an Auriga Fib-SEM (Zeiss, Oberkochen, Germany) as described previously [8, 21]. Probes were sputter-coated with gold for 1 min using an EMITECH-K550 sputter coater at an operating pressure of 7 × 10^2^ bar and a deposition current of 20 mA.

### Statistical analysis

Data are expressed as mean-standard deviation. Data normality was verified by the Shapiro-Wilk test. Results were statistically interpreted by one-way analysis of variance (ANOVA) with Bonferroni’s correction for multiple comparisons or Friedman test with Dunn’s correction for multiple comparisons, as appropriate. Comparisons between paired variables at different time points were done using repeated measures ANOVA with Tukey’s multiple comparisons test for post hoc analysis. Pairwise comparison between different co-cultures was done using unpaired t-test or Mann-Whitney test, as appropriate. Statistical analysis was performed using GraphPad Prism version 7.0a, La Jolla California USA.

A *p*-value ≤0.05 was considered statistically significant.

## Abbreviations

2D: Two-dimensional
3D: Three-dimensional
ADSC: Adipose tissue derived mesenchymal stromal cells
ADSC/Mb: Adipose tissue derived mesenchymal stromal cells co-cultured with myoblasts
bFGF: Basic fibroblast growth factor
BMSC: Bone marrow derived mesenchymal stromal cells
BMSC/Mb: Bone marrow derived mesenchymal stromal cells co-cultured with myoblasts
CK: Creatine kinase
DHS: Donor horse serum
FCS: Fetal calf serum
IGF: Insulin-like growth factor
Mb: Myoblasts
MSC: Mesenchymal stromal cells
PCL: Poly-ε-caprolacton
P/S: Penicillin/streptomycin
PRP: Platelet rich plasma
